# Supplementary material for: Are carbon emissions trading and green financial instruments synergistic? -Comprehensive quantitative research based on content analysis
Source: PLoS One. 2024 Mar 7;19(3):e0298601. doi: 10.1371/journal.pone.0298601 (PMC10919601; doi:10.1371/journal.pone.0298601)
Supplement: S1 Data — (ZIP) [file pone.0298601.s001.zip › Data/地级市Z_F工作报告.zip!/╔╜╢1⁄2/▒⌡╓▌╩╨/└┤╫╘╣1⁄2╓┌║┼í╛┬φ┐╦╩2╛▌═°í┐_2017.docx]

各位代表:

现在，我代表市人民政府向大会报告工作，请予审议，并请市政协各位委员提出意见。

一、2016年和本届政府工作回顾

过去一年，在以习近平同志为核心的党中央正确领导下，我们认真贯彻落实省委、省政府和市委决策部署，凝心聚力抓发展，较好地完成了各项目标任务，实现了“十三五”良好开局。地区生产总值实现2470亿元，增长7.2%；一般公共预算收入达到220亿元，增长8.2%；固定资产投资完成2157亿元，增长8.5%；规模以上工业增加值增长6.5%；进出口总额达到577亿元，增长14.8%；城镇、农村居民人均可支配收入达到30583元、13736元，分别增长7.7%、7.9%。

（一）经济结构进一步优化。坚持创新发展，预计全市研发经费占GDP比重达到2.6%，高新技术产业产值占比达到26.8%。坚决化解过剩产能，淘汰落后常减压炼油装置357万吨。积极稳妥推进“去库存”，房地产去化周期缩短至9个月，规模以上工业产销率达到102.2%。新增银行、保险、证券等金融机构8家，资本市场直接融资86.3亿元，增长28.8%。规模以上工业企业全员劳动生产率达到36.6万元，比上年提高2.3万元。着力补齐服务业发展短板，成功举办第二届中国古村镇大会，打造了魏集古镇、西纸坊村等一批旅游新亮点。投资12亿元的滨州万达广场建成开业。淘宝镇、村分别达到3个、22个，均占全省的四分之一，电子商务年交易额达1303亿元；服务业增加值实现1095亿元，占GDP的比重提高2.4个百分点，提升幅度全省第一。三次产业结构调整为9.4：46.3：44.3。

（二）农业生产能力稳步提高。粮食总产306万吨，实现丰产丰收。农林牧渔业总产值实现486亿元，增长5.6%。市级以上重点农业龙头企业达到381家，农民合作社、家庭农场分别发展到5358家、753家。黄河三角洲（滨州）国家农业科技园区通过科技部验收，沿黄生态高效现代农业示范区列入省“十三五”规划，“渤海粮仓”示范片区达到90个、面积33万亩。粮食生产“十统一”面积达到71万亩，完成农田节水灌溉13.5万亩，“粮改饲”推广面积达20万亩，农村耕地规模经营占比达38.8%。强化农业标准化建设，严格过程监管，农产品质量合格率达99%以上，“三品一标”认证产品412个，省级农产品质量安全市、食品安全城市创建积极推进。

（三）重大基础设施项目加快推进。滨州港边运营边建设，3万吨级液化码头完成主体，吞吐量达到375万吨。滨港铁路二期全线展开，已完成投资8.6亿元。交通路网建设取得积极进展，济滨东高速、滨惠大道、南外环道路扩建、东外环北延等4条道路同步通车。秦滨高速埕口至沾化段、长深高速高青至广饶段开工建设，沾化至临淄高速、沿海高等级公路等重点工程前期工作进展顺利。济青高铁邹平段、黄大铁路顺利推进。南水北调配套工程博兴、邹平供水单元全面建成。扎实推进“气化滨州”，新增用气指标5亿立方米。

（四）生态环境不断优化。全面完成中央、省环保考核指标任务。持续治理城市脏乱差，新立河水体、城区扬尘整治成效明显，顺利通过全国文明城市年度测评。成功申报省级海绵城市试点市，积极推进水生态文明城市创建，黄河岛、秦皇河国家湿地公园荣获“山东最美湿地”称号。积极推进节能减排，完成8个重点工业园区企业污水“一企一管”改造，51台10万千瓦及以上机组完成超低排放改造，秸秆禁烧工作成效明显。二氧化硫平均浓度改善31%，改善幅度列全省第2位；“蓝天白云”天数232天，较上年增加37天。中国社科院发布的《城市蓝皮书》中，我市位列全国28个健康发展城市第20位。

（五）改革开放取得新成效。加大简政放权力度，全面推行“五证合一”登记制度，各类市场主体达23万户，增长21.6%。积极优化金融环境，组建成立了滨州农商行，全面完成农信社银行化改革。成功举办第三届资本对接大会。改组成立市财金投资集团公司，设立市级资产管理公司和再担保公司。在全国地级及以上政府财政透明度排行中，我市居全国第15位、全省第1位。完成农村土地承包经营权确权颁证工作，位列全省优秀等次。作为全国公立医院改革试点城市，全市9家城市公立医院综合改革全面启动，药品加成全部取消。中小学去行政化和校长职级制改革基本完成。对外开放取得新成效，引进市外到位资金327亿元、增长21.5%。争取原油进口指标757万吨，占全省的13%。加快企业“走出去”步伐，新设境外企业16家，魏桥创业印尼项目一期工程建成投产，西王集团成功并购加拿大科尔公司。

（六）社会民生事业快速发展。全市一般公共预算民生支出262.4亿元，占财政支出的83%。精准扶贫成效明显，投入专项扶贫资金2.7亿元，实施扶贫项目878个，实现7.2万贫困人口脱贫、213个贫困村摘帽，超额完成年度脱贫任务。着力解决城镇中小学“大班额”问题，完成投资70.6亿元，开工项目572个、388万平方米，竣工358个、219万平方米。农村改厕50.8万户，完成年度计划的109%。市城区老旧小区改造步伐加快，棚户区改造新开工9660户、基本建成7745户，分别完成省任务的105%和172%。新增城镇就业4.2万人，农村劳动力转移就业6.3万人。常住人口城镇化率达到56.8%，提高2.2个百分点。创新养老服务模式，新建农村幸福院192处，新增养老床位2200张。积极推进农村公路安全生命防护工程，完成治理2851公里，位居全省前列。在全国率先编制完成市级《“十三五”军民融合深度发展规划》，“滨州舰”正式命名入列，实现创建全国双拥模范城“三连冠”。实施全面二孩政策，出生人口性别比下降3.8个比值。文体事业加快发展，现代吕剧《兰桂飘香》代表山东参加全国少数民族文艺会演，魏桥创业、八一京博控股两支俱乐部在全国乒超联赛中获得优异成绩，老年门球队夺得亚洲冠军。安全生产、社会治安不断强化，全市大局和谐稳定。

各位代表，2016年各项任务的完成，标志着本届政府工作顺利收官。过去的五年，全市上下始终坚持“六个更加注重”，砥砺奋进，积极作为，谱写了滨州科学发展、加快发展的新篇章。

这五年，我们始终坚持发展第一要务，经济实力大幅提升。全面落实稳增长政策措施，狠抓骨干产业引领发展，打造了高端铝产业、新型化工、粮食加工、家纺纺织四大千亿级产业集群，产值占全市规模以上工业的75%以上。积极建设汽车轻量化材料基地和高端轮毂制造中心，大力发展博兴厨具和板材、惠民绳网等区域特色产业。魏桥集团世界500强排名跃升至163位，4家企业入选中国企业500强，8家企业入选中国制造业500强，4家企业跻身全国粮油行业10强，粮食产业创造出了“滨州模式”。积极做好对上争取，国家专项建设基金项目、资金数量均居全省前列。主要产业产能列入工信部符合行业规范条件名单，纳入国家统计局统计范围，为加快科学发展奠定了坚实基础。五年来，全市地区生产总值增长650亿元，进出口总额增长145亿元，一般公共预算收入增长89亿元、总额列全省第10位，税收收入增长70.5亿元、总额列全省第9位。金融机构本外币存贷款余额分别达到2698亿元、2394亿元，连续五年增幅列全省前列，黄河三角洲中心城市的地位更加突出。

这五年，我们始终坚持打基础、利长远，重大基础设施建设实现历史性突破。深入实施“黄蓝”两区和省会城市群经济圈战略，持续加大开发力度，全力加快基础设施建设。滨州港通航，德大铁路、济滨东高速通车，开启了滨州交通历史上的新纪元。邹平经济技术开发区、滨州经济技术开发区升级为国家级开发区。作为全市新的经济增长极的北部沿海加快开发，北海经济开发区一般公共预算收入突破10亿元，产业新城加速崛起。

这五年，我们始终坚持统筹协调发展，城乡面貌发生深刻变化。在城区，完善提升“四环五海·生态滨州”，城区南环道路、南海一路改造通车，北环水系改造完成，建设黄河引水沉沙池，实现“清水入城”。加强水系和绿化景观建设，把秦皇河、新立河打造成了两条靓丽风景带。全面实施城区环境综合治理，改造农贸市场、拆除城区旱厕、治理露天烧烤、整顿乱停乱放，城区环境更加优美宜居，高标准通过了国家卫生城复审。在乡村，从2013年开始，前三年“村村修道路”，近两年“户户改厕所”，累计投入80多亿元，圆满完成所有村庄道路硬化任务；投入7.6亿元完成了65%农户的改厕任务，累计改造农村危房2.1万户。村庄净化、环境美化加快推进，“美丽乡村”初步展现，农村环境面貌发生了历史性巨变。

这五年，我们始终坚持民生为先的施政理念，让人民群众享受到更多改革发展成果。时刻牢记“小康路上一个都不能掉队”的指示要求，全面打响了“五年任务、两年完成”的脱贫攻坚战役，并取得首战告捷。高度关注养老事业和弱势群体，连续5年提高居民基础养老金、退休人员养老金标准，建成投用市社会养老服务中心，完成83处乡镇敬老院旱厕改造，全市养老床位新增1.8万张。设立特困家庭救助基金，2292户受到救助帮扶，连续三届入选中国慈善百强城市。大力实施保障性安居工程，惠及居民6.7万户。坚持“百年大计、教育为本”的方针，累计新建幼儿园135处，在全省率先实现校车全覆盖；全面启动解决城镇中小学“大班额”问题，是历史上教育投入最大的时期。努力改善基层医疗环境条件，新建910处村卫生室，全面实行乡镇卫生院住院起付线以上全报销。千方百计改善市民文化体育生活，市图书馆、博物馆、文化馆、科技馆和市民体育健身广场相继建成投用，群众生活质量明显提升。

这五年，我们始终坚持推进依法行政、廉洁从政，政府自身建设不断加强。认真贯彻党组工作条例，坚决服从市委领导，扎实开展党的群众路线教育实践活动、“三严三实”专题教育、“两学一做”学习教育。认真落实中央八项规定精神，持之以恒纠治“四风”，坚决惩治腐败，五年来立案3627件，给予党政纪处分3640人，移送司法机关65人。扎实推进“放管服”改革，市级行政许可事项由462项压减至321项，非行政许可事项全部取消，市场活力进一步激发，民营经济进一步发展，市场主体增长了161%。各级法治意识明显增强，地方立法实现零的突破，建立实施党政一体法律顾问制度，中介超市运行良好，市政府荣获首届中国“阳光政府奖”，积聚起了敢于担当、奋发进取的强大合力。

各位代表，回顾五年来的工作，成绩值得自豪。这是我们认真学习习近平总书记系列重要讲话精神，深入贯彻新理念新思想新战略的结果，是省委、省政府坚强领导、深切关怀的结果，是全市上下在市委统揽指挥下团结拼搏的结果。在此，我代表市人民政府，向为滨州发展付出心血汗水的全市人民，向给予政府工作有力支持的各界人士，向为滨州发展做出积极贡献的驻滨部队官兵、公安干警，向所有关心支持滨州发展的海内外朋友，致以崇高的敬意和衷心的感谢!

各位代表，在总结成绩的同时，我们也清醒地认识到，我市综合实力还不够强，稳增长任务依然艰巨；产业层次低、中低端产品占比大问题突出，企业创新能力整体较弱；民生保障能力还不够强，扶贫攻坚等工作任务仍然十分繁重；节能减排、环境保护、安全生产压力很大，食品安全、金融风险等方面隐患不容忽视；政府职能转变还不到位，人员素质、工作作风、办事效率、廉洁自律等方面，人民群众还有不满意的地方。对于这些问题，我们将采取有效措施，认真加以解决。

二、今后五年的主要目标和任务

未来五年是区域发展的关键时期，也是提前全面建成小康社会的决胜期。全市经济社会发展的总体要求是：全面贯彻党的十八大和十八届三中、四中、五中、六中全会精神，深入学习贯彻习近平总书记系列重要讲话精神，按照市第九次党代会确定的目标任务，以新理念新思想新战略为引领，坚持稳中求进工作总基调，坚持“六个更加注重”，以提高发展质量和效益为中心，以推进供给侧结构性改革为主线，着力稳定经济增长、深化改革开放、推动转型升级、防范化解风险、保障改善民生，全面建设更高质量更高水平的小康滨州。

按照上述总体要求，今后五年经济社会发展的主要预期目标为：地区生产总值年均增长7.5%左右，一般公共预算收入年均增长8%左右，规模以上工业增加值年均增长8%左右，固定资产投资累计实现1.5万亿元左右，社会消费品零售总额年均增长9%左右，外贸进出口总额增长平稳，城镇、农村居民人均可支配收入年均分别增长7%左右、8%左右。到2021年，经济社会发展跃上新台阶，区域发展的地位和作用更加突出。

（一）实现创新发展新突破。推动工业创新，加快培育壮大新动能，是塑造滨州竞争新优势、抢占发展制高点的迫切需要，要持之以恒地紧抓不放。要坚决落实好“三去一降一补”各项要求，坚决完成钢铁“去产能”任务，不再扩大电解铝产能。要大力振兴实体经济，切实抓好四大千亿级产业集群的提质增效发展，确保总量跨过“万亿大关”。要注重传统产业和新产业融合发展，着力提高新一代信息技术、高端制造、生物、绿色低碳、数字创意五大新兴产业占比。深入实施“互联网+”行动计划，积极推进工业化、信息化深度融合，加快推进军民融合发展。扎实推进农业供给侧结构性改革，加快现代农业产业体系、生产体系、经营体系建设，持续提高农业生产能力。积极发展“新六产”，提高农民在一二三产业融合发展中的收益。大力发展现代服务业，努力扩大规模、提高质量，优化三次产业结构，实现经济发展方式的深度转变。

（二）实现区域协调发展新突破。今后五年是加快区域发展、加快重大基础设施建设的历史机遇期，要深入推进“黄蓝”两区战略实施，更深入地对接京津冀、融入省会城市群经济圈，落实山东半岛城市群发展规划。加快构建更加快捷的交通体系，积极推进快速铁路和高速公路建设，争取尽快开工济滨城际铁路、京沪沿海通道，建设形成市城区“环城高速”路网。围绕“北部沿海产业新城”目标，加快港口建设发展，建成5万吨级深水航道，增加泊位运行，初步达到国家中型港口规模。建成运营滨港铁路、沿海高等级公路，吸引产业、资金和人才向北部沿海聚集，全力打造新的经济增长极。建设滨州黄河第四大桥，加快主城区跨河发展。实现小清河复航，带动沿线建设开发。支持重点区域创新发展，打造沿黄生态高效现代农业示范区，推动省级以上各类开发区突出主业、加快发展。

（三）实现生态建设新突破。坚持绿色发展，大力实施节能降耗，发展高效清洁能源，推广煤炭清洁化利用，消化能耗存量、严控能耗增量，确保完成节能目标。持续推进大气质量改善，严格控制大气污染物排放总量，积极应对重度雾霾天气，到2021年环境空气质量优良天数达80%以上。加强重点流域水环境和城市河道整治，持续推进水质改善。实施生态湿地保护与修复，加强近海海域环境保护，全面推进生态海洋建设。持续推进土壤污染治理，探索推行固废资源化利用。做好生态与旅游结合文章，倡导绿色文明生活方式和消费模式，齐心协力建设天更蓝、山更绿、水更清的良好环境。

（四）实现改革开放新突破。持续释放改革红利，大力推进简政放权，推行综合执法改革，全面构筑社会信用体系，营造法治化的营商环境。完成财税体制改革目标任务，用市场化手段运作好产业基金，健全完善债务风险防控、应急处置机制。加快金融改革创新，保持存贷款规模稳定增长，逐步降低担保贷款比重，积极推 动多层次资本市场融资，创造良好金融环境。稳妥推进农村土地所有权、承包权、经营权“三权分置”，稳步推进农村集体产权制度改革。加快文化教育、医疗卫生体制改革，建立健全公立医院、科研院所法人治理结构。认真研究应对贸易保护壁垒，鼓励企业“走出去”“引进来”，主动融入“一带一路”战略，积极参与国际市场和资源的开发利用，全面提升开放型经济水平。

（五）实现民生保障新突破。持续加大民生投入，全面完成脱贫任务，积极探索解决相对贫困和统筹解决城乡贫困问题。鼓励大众创业、万众创新，每年新增城镇就业3万人以上，城镇登记失业率控制在4%以内。加快推进新型城镇化，引导更多农民进城落户，常住人口城镇化率达到62%以上。完成解决城镇中小学“大班额”、义务教育“全面改薄”等重点工程，基本实现教育公共服务标准化、均等化。加强“健康滨州”建设，积极推行分级诊疗，完善城乡基本医疗保障制度。持续优化出生人口性别比，促进人口均衡发展。优化市级医疗资源布局，建成投用市人民医院西院区、妇幼保健院和中医院新院，完成精神卫生中心搬迁改造。保障妇女儿童权益，健全扶残助残服务体系。鼓励社会力量兴办教育、卫生、体育、养老等事业，基本建成覆盖城乡、发展均衡、充满活力的现代公共服务体系。强化安全红线意识，狠抓安全生产监管，严厉打击违法犯罪，确保社会大局和谐稳定。

三、2017年工作安排

今年是新一届政府开局之年，经济社会发展主要预期目标为：地区生产总值增长7.5%左右，一般公共预算收入增长8%左右，规模以上工业增加值增长8%左右，固定资产投资增长11%左右，社会消费品零售总额增长9%左右，进出口总额增长平稳，城镇登记失业率控制在3.6%以内，城镇、农村居民人均可支配收入分别增长8%左右和8.5%左右，人口自然增长率控制在14.6‰以内。完成国家和省下达的节能减排约束性指标。

实现上述目标，要着重做好以下几方面的工作：

（一）抓好实体经济发展。实现稳中求进、振兴实体经济的关键在工业，突出强化四大千亿级产业集群的“四梁八柱”作用，带动汽车轻量化、商用厨具、金属板材、化纤绳网等特色产业基地和中小企业发展。要坚持规划引领，围绕深化供给侧结构性改革，高标准编制产业发展规划，实施分类指导。坚定不移化解过剩产能，对电解铝、化工等重点行业，要由量的增长转向质的提高，不断拉长产业链条，提高附加值，增加产出效益。要强化政策支持，发挥政府引导基金作用，吸引社会和金融资本设立产业基金，定向支持实体经济投资项目，用好再担保机制帮扶中小企业，执行好各项减税降费政策，完善基础设施配套服务，切实为企业“去杠杆”、降成本、减负担、增活力。要突出科技支撑，深入实施创新驱动战略，支持企业与科研院所、高等院校开展合作，加快新材料、新能源、电子信息、生物医药等新兴产业发展。实施知识产权强市战略，鼓励企业自主创新，积极开展技术改造、技术攻关，提升实体经济的创新能力。加大引才引智力度，鼓励企业专注擅长领域，进行深耕细作，创建驰名商标，打造自主品牌，培育更多“百年老店”。巩固树立标准意识，支持企业发扬工匠精神，积极主导或参与制定国家、国际标准，增强市场话语权。要大力招商引资，围绕主导产业上下游配套，积极争取与世界500强企业开展合作共赢，主动承接京津冀地区产业转移。充分发挥产业协会作用，加强骨干企业与国内外大型企业对接合作。加快推进美国江森启停电池、河北立中集团卡巴（卡车、大巴士）铝合金轮毂、开发区高端铝材精深加工产业园等重点项目建设，续建、开工投资5000万元以上工业项目224个。要落实区域责任，把四大千亿级产业集群发展目标，分解落实到各个县区和重点企业。加快重点区域开发，强化省级以上开发区招商引资和项目建设主战场作用，搞好运行机制改革创新，打造率先发展新高地。要加强企业家队伍建设，培养引进有国际视野的职业经理人、市场总监、财务总监，建立现代企业制度。要鼓励企业放心投资、大胆创业，主动为企业排忧解难，构建“亲”“清”新型政商关系。严格落实企业主体责任，切实做到安全生产、诚信守法、绿色发展。

（二）抓好农业供给侧结构性改革。要重点研究市场需求，围绕城乡居民消费，谋划好农业生产，不断增加绿色优质农产品供给。持续优化产品结构，不断提高粮肉果蔬渔的品质和效益，粮食生产上，发挥泰裕麦业等龙头企业带动作用，积极推广专用优质品种，做强粮油加工品牌，放大“滨州模式”效应。肉类生产上，支持鸿安、亿利源等重点企业发展，扩大“粮改饲”面积，壮大养殖规模，扩大品牌影响。林果生产上，围绕沾化冬枣、阳信鸭梨等特色产品，研发新优品种，稳定提升产品质量，打造绿色、优质名牌。蔬菜生产上，依托沿黄生态高效现代农业示范区，发挥土壤水肥优势，扩大标准化种植面积，提供更多放心优质菜品。渔业生产上，重点抓好沿海、沿黄高效养殖带发展，推进“海上粮仓”建设。巩固改善农业生产条件，继续推进林水会战，完成小开河灌区、簸箕李灌区节水改造，重建徒骇河廿里堡闸，积极推进小农水重点县项目，发展高效节水灌溉24万亩。多引多蓄黄河水，增强防汛抗旱能力。抓好交通干线绿化长廊建设，改造农村危桥100座、农村电网1624公里。抓好农业生产服务，强化科技创新推广，加快黄河三角洲（滨州）国家农业科技园区建设，抓好耕地质量提升和“十统一”面积增加，研究政策支持，开发、建设好“渤海粮仓”示范工程。推动农业“接二连三”，配套发展保鲜贮藏、精深加工、物流配送等项目，提高农产品网上销售比重，实现农业“新六产”跨越式发展。发展适度规模经营，稳妥推进农村土地流转，农村土地所有权、承包权和经营权分置取得重要进展，新发展一批有实力的农业龙头企业，着力培育新型市场主体。

（三）抓好服务业壮大提升。大力发展“互联网+”，围绕生产生活、产品销售和售后服务，抓好“互联网+”规划策划。深化与京东、华为等企业的合作，加快黄河三角洲云计算大数据产业基地建设，将云计算、大数据的技术优势与我市特色产业资源优势相结合，细分行业市场，精准定位消费群体，实现工业产品的个性化定制和消费产品的个性化供应，促进服务业“以高补晚”。精培优选一批地理标志产品，进入大型网络销售系统，进入冷链物流。加快推进跨境电商发展，打造特色外贸小镇，规范发展快递配送，电子商务交易额突破1700亿元。做大做强现代服务业，围绕社会发展、消费需求，大力发展健身养生、居家养老、文化娱乐、教育培训等生活性服务业。加快批发零售、住宿餐饮业转型升级，实施智慧便利店进社区工程，推动中裕食品、三利快餐等连锁品牌发展壮大，努力打造15分钟居民便利生活圈。积极发展乡村旅游，合理规划景点、路线，加快推进全域旅游发展。着力发展金融服务业，进一步增强金融服务支持地方经济社会发展能力，加大金融对实体经济支持力度。积极开发小额贷、助保贷、税易贷等金融创新产品，加快普惠金融发展，为中小微企业提供良好服务。支持重点服务业项目建设，发挥万达广场建设运营示范效应，加大城市综合体、服务业旗舰品牌引进力度。强化京博物流等骨干企业引领作用，科学规划、打造提升一批现代物流园区和物流大市场。

（四）抓好城乡统筹发展。坚持一张蓝图干到底，做好新一轮城市总体规划修编，实现城市总体规划、经济社会发展规划、土地利用规划、生态环保规划“多规合一”，推进控制性详细规划全覆盖。开展城市设计，提高城市规划建设水平。加快推进城中村、棚户区改造，把城中村、棚户区改造作为城市工作的重中之重，抓住国家支持这方面融资贷款的政策机遇，大力实施集中改造。要让利于民，不求经济上挣钱，但求人民群众生活条件改善、城市面貌改变。积极推进商品房“去库存”，强化供需双向调节，突出“控量稳价”，逐步解决商业用房结构性库存问题。要加大老城等重点项目改造建设力度，对老城区部分道路路口进行升级改造，提高道路通行能力，继续改造打通部分“断头路”。推进海绵城市试点，建设地下综合管廊16.8公里。做好北环河林带提升和南环水系治理，加快城市水系连通工程建设。强化新立河、秦台河污水排放监管，治理张肖堂干渠、码堡沟等黑臭水体，确保达到全国水生态文明城市验收条件。提高城市精细化管理水平，进一步改革理顺城市管理体制，深入开展城市违法建设治理行动，着力解决乱搭乱建、道路扬尘、乱停乱放等问题，严格城市管理，巩固好国家卫生城复审成果。扎实推进新型城镇化，进一步放宽进城落户条件，实现5.9万农业转移人口市民化，加快5万城中（边）村居民完全市民化，把非农经济和就业超过70%的村庄和人口纳入城镇管理。加快“气化滨州”建设，基本实现天然气“镇镇通”。积极推进美丽乡村建设，“两年改厕”的任务必须全面完成，实现农村无害化卫生厕所全覆盖。研究解决畜禽养殖粪污处理和综合利用，抓好试点，逐步推开。提升美化、净化、亮化水平，逐步让广大农村群众过上同城里人一样的健康文明生活。

（五）抓好重大基础设施建设。加快港口开发建设，建成2个3万吨级液体化工泊位，新开工2个5万吨级油品泊位，加快推进5万吨级航道前期工作。加快滨州港至中海沥青输油管线建设。建成通关服务中心，争取滨州港口岸对外开放获得国务院正式批复，海港港区吞吐量突破1000万吨。建设环城高速路网，以长深高速为西环、济滨东高速为北环，依托长深高速高青至广饶段为南环、沾化至临淄高速为东环，搭建起市城区高速外环框架。秦滨高速埕口至沾化段、济青高速改扩建完成路基桥涵工程。规划建设滨海快速通道。加快铁路工程建设，完成滨港铁路7亿元投资任务，推动黄大铁路、济青高铁邹平段等工程建设，争取济滨城际铁路年内开工，全力做好京沪沿海通道规划建设的协调争取。加快沿海高等级公路、滨州黄河第四大桥、乐安黄河公路大桥等项目前期工作，确保小清河复航工程年内开工。抓好能源项目建设，规范新能源项目管理，做好对上争取，完成第一批20万千瓦光伏电站项目建设。

（六）抓好生态环境建设。加大节能减排力度，严格落实“去产能”要求，强化安全、环保、能耗倒逼机制，依法推进高耗能、高污染、低效益企业的关停并转。加强耕地保护，提高土地集约节约利用水平。抓好环保突出问题整治，打好大气、水、土壤污染防治三大战役，推进燃煤机组超低排放改造，市县城区全部淘汰10蒸吨及以下燃煤锅炉。全面实施秸秆禁烧，推动综合利用。全面推行河长制，强化流域治污，实现6个国控断面全指标考核达标。加大对第三方管理运营污水处理厂的监管力度，确保水质稳定达标。抓好土壤污染防治任务落实，加强危险废物规范化处置管控。全面实施“工业绿动力”计划，实行能耗强度和能耗总量“双控制”，明确县区任务，落实企业责任，控制能耗增长过快势头。加快推进散煤清洁化治理和农村清洁取暖试点。构筑生态保护体系，加强资源环境生态红线管控，严格执法监管，重视环境信访问题，统筹建设生态环境监测网络，对重点流域、重污染天气实行联防联控联治，提高重度雾霾等极端天气的应急响应能力。

（七）抓好改革开放。稳妥推进财税体制改革，严格预决算公开规程，进一步增强财政透明度，加强政府购买服务、政府投资预算评审、项目代建管理。明确区分经常性支出和建设性支出，财政预算资金优先保证工资、津贴补贴等经常性支出；对建设性资金需求，多途径开辟融资渠道，采用政府购买服务、PPP等模式，引导社会资本参与公共服务事业和基础设施项目投资、建设与运营。加快金融领域改革，着力推进“去杠杆”，引导银行回归主业、回归实体经济，实施差异化信贷政策，支持企业市场化、法治化债转股。加大直接融资力度，促进企业主板、创业板、新三板上市挂牌。依法严厉打击逃废银行债务行为，用足用好贷款核销等政策空间，下决心处置一批风险点。充分发挥再担保公司和县区政府控股担保公司作用，加快资产管理公司改造为混合所有制企业，加大解决企业间相互担保“破圈断链”的力度。全面深化重点领域改革，加快推进综合行政执法体制改革，建立市县乡层级明确的执法体系。深化医保支付方式改革，建成全市医保统一结算平台。巩固扩大“五证合一、一照一码”改革成果，深化工商登记便利化改革，激活促进民营经济发展。加大国有资产监管改革力度，开展市属经营性国有资产统一监管，减少对一般性企业参股，积极推进国有企业“瘦身健体”。积极推进对外开放，加快发展外贸综合服务平台、跨境电商、海外仓等新兴贸易业态，培育出口支柱企业。鼓励企业开展国际产能合作、海外并购，开设海外生产线、工厂和产业园，扩大相关产品出口和重要资源进口。加大招商引资力度，扩大利用外资规模，开拓新的合作领域。四大产业集群各重点企业，要强化沟通协调，加快优势产品“抱团出海”。

（八）抓好民生实事。要坚决打赢脱贫攻坚战，加大资金投入，统筹整合涉农资金，发挥金融扶贫作用，加快推进产业扶贫、行业扶贫、社会扶贫，深化企业扶贫行动，全面完成1.8万建档立卡贫困人口脱贫任务。建立巩固脱贫成果长效机制，确保贫困人口及时纳入、精准退出，防止返贫、复贫。抓住机遇，力促教育工作上台阶，解决城镇中小学“大班额”问题，两年投入120多亿元，亘古未有，机遇难得，必须乘势而上，保质保量完成。坚持以立德树人为根本，继续深化基础教育综合改革，实施质量提升工程。积极探索集团化办学和高中阶段学校混合所有制办学模式，大力推进社会力量办学。支持滨州学院拓展航空专业、滨州职业学院创建优质高职院校。提高社会保障水平，深入实施全民参保登记计划。鼓励引导企业、社会团体和个人积极参与慈善救助。提高社会救助标准，建立城乡统一的特困人员救助供养制度。完成74处敬老院的消防设施提升改造。努力提高公共服务水平，开工建设市人民医院西院区、妇幼保健院和中医院新院，提升完善市社会养老服务中心功能。启动“健康滨州”建设，编制实施《健康滨州2030规划》和实施方案。加强食品药品安全监管，完成市食品实验室扩建工程，积极创建省级食品安全城市。基本完成村（社区）综合性文化服务中心建设，滨州大剧院建成投用，提升档案馆建设水平，加强“扫黄打非”和文化市场监管。继续推进“15分钟健身圈”建设，加快市民体育健身广场升级扩建，提高各类体育场馆利用率。全力做好民族宗教工作，深入推进双拥共建。倡导社会主义家庭新风尚，积极创建全国文明城市。加强社会综合治理，积极推进公共法律服务体系和法律教育基地建设，全面推行“一村一顾问”制度。严打严处各类违法犯罪，完善推广“一村一警”社区警务模式，提高禁毒和反恐工作水平。始终把安全生产作为生命线、高压线，深入落实用于安全生产的时间、资金、人员、程序“四个不能省”，坚决遏制重特大安全事故发生。健全完善社区治理服务体系，高度关注群众信访、司法建设等工作，保持社会和谐稳定。

四、加强政府自身建设

面对新的形势要求，各级政府要进一步增强政治意识、大局意识、核心意识、看齐意识，自觉维护以习近平同志为核心的党中央权威，坚决贯彻落实上级党委政府决策部署，确保政令畅通、令行禁止，以奋发有为的精神状态推动各项工作。

（一）坚持担当有为。时刻牢记人民政府为人民的宗旨，坚持主动作为，切实履行职责，自觉肩负起加快赶超的发展重任。努力克服本领恐慌、素质短板，大兴学习调研之风，加快知识更新，优化知识结构，持续推进境界提升、标准提升、素质提升。加强政务督查和效能监察，狠抓工作落实，认真整肃懒政怠政行为，决不允许有令不行、有禁不止。大力弘扬敢想、敢干、敢当的创业精神，定了的事情必须抓紧实施、及早完成，部署的工作必须善始善终、善作善成。要健全完善容错纠错机制，旗帜鲜明地为担当者担当，对负责者负责，让优者上、庸者下、劣者汰，引导广大干部积极投身招商引资、项目建设、脱贫攻坚等工作一线，努力营造风清气正、激情干事的浓厚氛围。

（二）坚持透明高效。持续推进简政放权、放管结合、优化服务，进一步减程序、降费用、缩时限，切实提高服务的质量和效率。编制公共服务事项目录，向社会公开发布，推进政务公开标准化、规范化。更多引入市场机制和市场化手段，减少政府对资源的直接配置，让企业和个人有更多活力和更大空间去发展经济、创造财富。积极推进政府管理与信息化融合，加快大数据中心建设，完善市县两级政务服务平台，推动公共资源交易改革创新，让数据多跑路，让群众少跑腿，让群众办事创业更方便、更快捷、更舒心。

（三）坚持依法履职。自觉运用法治思维和法治方式推动工作，把依法行政贯穿于政府工作的各个层面、各个环节。认真落实重大行政决策法定程序，自觉接受人大及其常委会的法律监督和人民政协的民主监督，主动接受舆论监督，密切与各民主党派、无党派人士和人民团体的联系沟通。推进审计监督全覆盖，强化对民生领域、重点投资项目的审计力度，确保中央和省、市委决策部署落实到位。深入推行政府法律顾问制度，依法稳妥化解各类矛盾纠纷，维护群众合法权益。

（四）坚持反腐倡廉。严格落实全面从严治党政治责任，强化守纪律、讲规矩要求，深入推进从严治政。加强权力运行监督，狠刹歪风邪气，坚决制止吃拿卡要行为，决不允许任何单位、任何公务人员向基层和企业“乱伸手”。深入落实党风廉政建设责任制和“一岗双责”，标本兼治、反腐倡廉，加强对政府部门领导干部的廉政教育、日常管理和监督约束，严肃查处发生在重点领域、关键环节和群众身边的腐败案件。持之以恒落实中央八项规定精神，坚决反对“四风”，厉行勤俭节约，严控“三公”经费，打造崇德尚廉、遵纪守法的公务员队伍。

各位代表，站在新的历史起点上，面对时代赋予的重任、人民寄予的厚望，我们深感责任重大、使命光荣。让我们紧密团结在以习近平同志为核心的党中央周围，在市委的正确领导下，奋发进取，主动作为，推动全市各项工作“争一流、上水平”，全面建设更高质量更高水平的小康滨州，以优异成绩迎接党的十九大胜利召开！
